# Information search under uncertainty across transdiagnostic psychopathology and healthy ageing

**DOI:** 10.1038/s41398-024-03065-w

**Published:** 2024-09-03

**Authors:** Greta Mohr, Robin A. A. Ince, Christopher S. Y. Benwell

**Affiliations:** 1 School of Psychology and Neuroscience, University of Glasgow, Glasgow, UK; 2 Division of Psychology, School of Humanities, Social Sciences and Law, University of Dundee, Dundee, UK

**Keywords:** Human behaviour, Depression

## Abstract

When making decisions in everyday life, we often rely on an internally generated sense of confidence to help us revise and direct future behaviours. For instance, confidence directly informs whether further information should be sought prior to commitment to a final decision. Many studies have shown that aging and both clinical and sub-clinical symptoms of psychopathology are associated with systematic alterations in confidence. However, it remains unknown whether these confidence distortions influence information-seeking behaviour. We investigated this question in a large general population sample (N = 908). Participants completed a battery of psychiatric symptom questionnaires and performed a perceptual decision-making task with confidence ratings in which they were offered the option to seek helpful information (at a cost) before committing to a final decision. Replicating previous findings, an ‘anxious-depression’ (AD) symptom dimension was associated with systematically low confidence, despite no detriment in objective task accuracy. Conversely, a ‘compulsive behaviour and intrusive thoughts’ (CIT) dimension was associated with impaired task accuracy but paradoxical over-confidence. However, neither symptom dimension was significantly associated with an increased or decreased tendency to seek information. Hence, participants scoring highly for AD or CIT did not use the option to information seek any more than average to either increase their confidence (AD) or improve the accuracy of their decisions (CIT). In contrast, older age was associated with impaired accuracy and decreased confidence initially, but increased information seeking behaviour mediated increases in both accuracy and confidence for final decisions. Hence, older adults used the information seeking option to overcome initial deficits in objective performance and to increase their confidence accordingly. The results show an appropriate use of information seeking to overcome perceptual deficits and low confidence in healthy aging which was not present in transdiagnostic psychopathology.

## Introduction

When making decisions in everyday life, we often lack immediate feedback about whether the decisions we have made are the right ones. Instead, we must rely on an internally generated sense of confidence to help us revise decisions and direct future behaviours [1, 2]. Subjective confidence provides an internal evaluative signal indicating the probability of a decision being correct [3, 4]. Recent studies have shown that confidence directly informs whether further information should be sought prior to commitment to a final decision, with states of low confidence being associated with increased information-seeking behaviour independently of initial accuracy [1, 5–9]. In other words, when individuals are unsure of the correctness of a decision, they seek further information to improve their decision-making [10]. Given this relationship, how is information-seeking behaviour affected by distortions of confidence? Systematic under-confidence may result in increased and inefficient information-seeking, and over-confidence may result in reduced information-seeking and the maintenance of incorrect choices, but there is currently little evidence directly addressing this question.

Numerous studies have shown that both clinical [11–14] and sub-clinical [13, 15–17] psychopathology are associated with selective alterations in metacognition and subjective confidence. For example, Rouault et al. [17] found that a transdiagnostic symptom dimension characterised by ‘anxious-depression’ (AD) was associated with systematically low confidence during performance of a perceptual decision-making task with confidence ratings, despite showing no detriment in objective accuracy. Conversely, a symptom dimension characterised by ‘compulsive behaviour and intrusive thoughts’ (CIT) was associated with systematic over-confidence. We recently replicated and extended these findings to show that the under- and over-confidence associated with AD and CIT, respectively, are reliable and domain general [15]. However, it remains unknown whether these confidence alterations influence information-seeking behaviour. Intriguingly, previous studies suggest that distortions of confidence associated with individuals holding radical and/or dogmatic political beliefs result in sub-optimal information-seeking under uncertainty [8, 18].

In addition to psychopathology, distortions of metacognition and subjective confidence have also been associated with healthy aging [19–21]. In a study by McWilliams et al. [20], older adults reported systematically low confidence across both memory and perception tasks despite showing no detriment in accuracy (i.e., performance comparable to young adults). This suggests an increase in negative confidence bias across the lifespan even when objective performance remains stable.

Here, we investigated the relationships between transdiagnostic psychiatric symptom dimensions, age, and information-seeking in a large general population sample (N = 908). Participants completed a short battery of psychiatric symptom questionnaires [22] and performed a perceptual decision-making task with confidence ratings in which we manipulated the cost of seeking information on each trial. We hypothesized that the anxious-depression dimension would be associated with systematically low confidence (but not reduced accuracy) and increased information seeking, whereas compulsivity would be associated with systematic over-confidence and reduced information seeking. Additionally, we hypothesized that age would be negatively related to subjective confidence and positively related to information seeking.

## Methods

### Participants

Participants were recruited online using the Prolific (https://www.prolific.co) and Sona Systems (https://www.sona-systems.com) recruitment platforms (1156 participants (874 Female/ 273 Male/ 9 Other), 17–80 years old (M = 33.32, SD = 14.73)). Some participants (N = 700) were paid £6 for their time, whilst others received undergraduate course credits (N = 456). All participants were offered a £20 reward if they achieved the top score on the information seeking task to motivate performance. The sample size was chosen to ensure adequate statistical power to detect effects at least as strong as those observed in a previous study from our lab [15]. Specifically, we based the power analysis (performed in G*Power) on the lowest significant effect size observed for a single symptom dimension across the symptom dimension-behaviour relationships in the previous study (Compulsive Behaviour and Intrusive Thought (CIT)-accuracy (*d’*) relationship: f^2^ = 0.02). The power analysis indicated that 395 participants would be required to achieve 80% statistical power to detect such an effect. While we had directly relevant previous work on which to base estimates of effect sizes for relationships between symptom dimensions and first-order accuracy and confidence, we had less to go on regarding potential symptom-information seeking relationships. In Schulz et al. [8], some participants didn’t use the information seeking option at all and so we assumed we may need a larger sample to detect relationships with this measure. Hence, the large sample size allowed for high statistical power to be retained, even after data exclusion.

Pre-defined exclusion criteria (outlined below) led to the exclusion of 314 participants, leaving a final sample of 908 participants (692 female/ 216 male aged from 17 to 80 years (*M* = 34.45, *SD* = 15.10)). The overall exclusion rate (21%) is in line with previous similar online studies both from our own group [15] and others [8, 16, 17, 23]. To ensure that the key results were not influenced by the exclusion of these participants, we repeated the key analyses on the full sample with no performance-based exclusions (Supplementary Fig. S5). The study received ethical approval from the University of Dundee Research Ethics Committee and University of Glasgow Ethics Committee (300210261). All participants provided informed consent. All methods were performed in accordance with the relevant guidelines and regulations, ensuring compliance with established standards.

### Perceptual information-seeking task

**Fig. 1:**
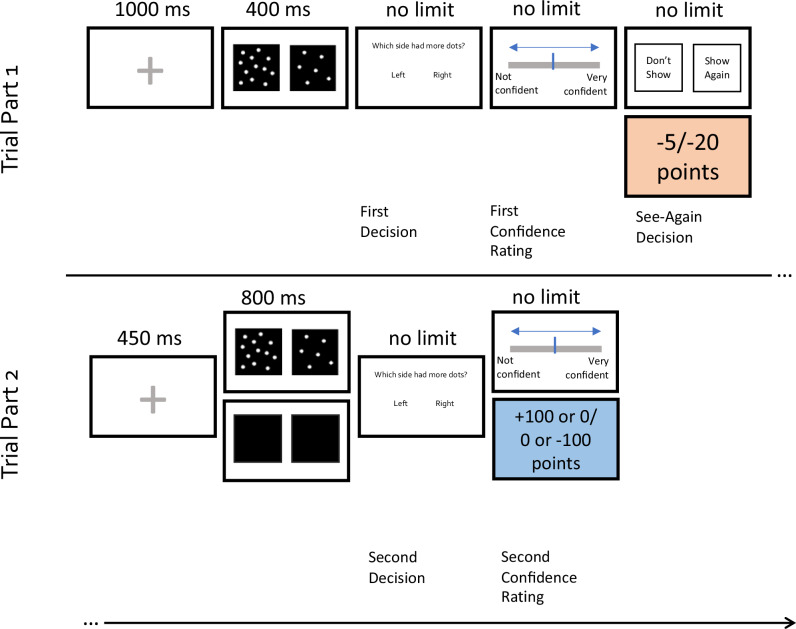
Information-seeking task. On each trial, participants judged which box (left or right) contained the higher number of dots and provided a confidence rating in each decision (scale of 1–6, where 1 represented “not confident (guessing)” and 6 represented “certain”). Participants were then given the chance to see the stimuli again for a certain cost and were subsequently shown either the stimuli or two blank black squares again. Participants were asked again to judge which box (left or right) contained the higher number of dots and provided a confidence rating. A correct final answer either provided a reward or the aversion of a loss.

The task was designed to combine the task parameters of Benwell et al. [15] and Schulz et al. [8]. A 2-alternative forced-choice numerosity discrimination task was used in which participants judged which of two boxes contained a higher number of dots. Figure 1 shows a schematic of the trial procedure. On each trial, a black cross appeared in the centre of a white screen for 1000 milliseconds (ms). This was followed by two black boxes, one on the left and one on the right side of the screen, which appeared simultaneously for 400 ms. Both boxes contained numerous white dots. The participant was asked to choose which box had contained a larger number of dots by pressing the ‘w’ key for left box or the ‘e’ key for right box. One box (the reference box) always contained 272 dots (out of 544 possible dot locations) while the other box contained an increased or reduced number of dots ranging from –64 to +64 dots (in increments of 8) in comparison to the reference. The location (left or right) of the reference box varied across trials and within each of the difficulty levels. The order of stimulus presentation was randomly generated for each participant within each block. Participants could respond after the stimulus screen changed to the response screen (i.e., not during the 400 ms of stimulus presentation). There was no time limit for the response and participants were not given feedback on whether their response was correct. After providing a response, participants were asked to rate ‘how confident are you that the decision was correct?’ on a scale of 1 (Not confident) to 6 (certain), equivalent to a half-confidence scale. Participants were instructed that if they were *very unsure* in their decision that they should choose the lowest (‘Not confident (guessing)’) option. There was no time limit for the confidence rating.

To assess the tendency to seek further information in cases of uncertainty, after the first choice and confidence rating the participant was offered the chance to see the stimuli again (for a cost of either -5 or -20 points, depending on the block). If they chose to see the stimuli again, they were presented the same stimuli for a further 800 ms. Hence, seeing the stimuli again was always helpful. If they chose not to see again, they did not lose any points, but they instead saw two empty boxes for 800 ms. Regardless of whether they sought further information or not, the participant then made a final judgement on which box had contained more dots and provided a final confidence rating. To incentivize subjects to strive for the best possible overall accuracy, they either gained (appetitive blocks) or lost (aversive blocks) points based on their performance on this final decision. In appetitive blocks, if they were correct in this 2^nd^ choice then they were awarded 100 points, whereas if they were wrong, they gained 0 points. In aversive blocks, if they were correct in the 2^nd^ choice then they lost 0 points, whereas if they were wrong, they lost 100 points. Participants were reminded of the current rules (i.e., appetitive v aversive, low versus high information seeking cost) on each trial. Each participant completed a total of 128 trials (16 trials at each difficulty level, split evenly into 4 blocks consisting of 32 trials each). Participants were instructed before beginning the task that the individual who achieved the highest number of points overall would receive a £20 reward on top of their initial renumeration for participation.

The rationale for including the cost manipulation was to replicate the task structure of Schulz et al. [8], who included high- and low-cost conditions. As Schulz et al. describe, a Bayes-optimal agent will seek information when the cost is outweighed by the likelihood of having made a mistake (indexed by uncertainty). As such, information seeking should be lower when the cost of seeking is higher. Therefore, including the cost manipulation in our study allowed us to ensure that participants were completing the task as expected, and provided consistency with Schulz et al. [8]

The rationale for the inclusion of the appetitive vs aversive blocks was to allow to us to further explore information-seeking behaviour by establishing a baseline for a deviation from optimal information seeking in the form of a potential loss aversion bias – i.e., would people be more willing to invest more in information seeking when avoiding a loss compared with earning a reward? [24]. Answering this question would inform models of metacognition and information seeking, e.g., to provide the potential to extend process models of metacognition to account for information seeking decisions and/or provide useful data for more detailed models of information seeking [9].

Participants could take a self-paced break between blocks. Before starting the task, participants completed 4 practice trials in which they only had to make the initial decision (stimuli presentation, response, confidence rating) with feedback (a green tick for correct or a red cross for incorrect). Two further practice trials were used to familiarise participants with the confidence rating scale in which they were instructed how to respond if they were confident or not confident. Participants then completed 6 practice trials which had the same structure as the main task trials (stimuli presentation, response, confidence rating, see-again decision, stimuli presentation, response, confidence rating) but participants could see their cumulative score on the screen throughout these trials. Participants were not informed of their score at any point throughout the main task. To counterbalance the order of information cost conditions, participants were randomly assigned to complete the experiment in either order A (appetitive/low-cost block; aversive/low-cost block; appetitive/high-cost block; aversive/high-cost block) or order B (appetitive/high-cost block; aversive/high-cost block; appetitive/low-cost block; aversive/low-cost block). Reward conditions were always presented with the appetitive block first to induce a stronger feeling of loss when previously gained points were deducted for incorrect responses [25, 26].

### Task outcome measures

For each participant we calculated 5 primary outcome measures. For each of the first and final decisions we calculated 1^st^-order accuracy (indexed by Type-1 sensitivity (**d’**)) and confidence (indexed by mean confidence rating across trials). We also considered the percentage of trials in which participants selected the information seeking option to see the stimulus again between the first and final decisions.

Note that due to recently reported limitations of the m-ratio [27] as a reliable and unbiased measure of metacognitive ability, particularly when tasks have under 400 trials [12, 28, 29], we did not include this measure as one of the primary outcomes. However, for completeness and consistency with previous studies [15–17], we report regression results between m-ratio and both age and symptom dimensions in Supplementary Fig. S6.

### Self-report psychiatric symptom questionnaires

Each participant completed selected items (total = 63) from a battery of eight mental health questionnaires [30–37] which assessed symptomology across a range of disorders. The items were identical to those used by [22] who showed that they provide an accurate approximation of three transdiagnostic symptom dimensions identified in previous research labelled ‘Anxious-Depression’ (AD), ‘Compulsive Behaviour and Intrusive Thoughts’ (CIT) and ‘Social Withdrawal’ (SW) [15, 17, 38]. To calculate dimension scores for each participant, the raw responses for each item were first z-scored across participants, then the individual item z-scores within each participant were multiplied by their corresponding factor weights [22] and the resulting products were summed across all items for each factor. Finally, the factor sums were z-scored across participants in preparation for statistical analyses.

### Procedure

The experiment was conducted online via the Gorilla experiment platform [39] and could only be completed on either a laptop or computer (and not on a mobile phone or tablet) to facilitate a more optimal screen size for the task. Participants were first directed to an information sheet and consent form. If informed consent was given, participants were asked to provide demographic information of age and sex assigned at birth. The participants then completed the questionnaires and task in a randomised order. The experimental session took approximately 1 h.

### Exclusion criteria

Several predefined exclusion criteria were applied to the data. Approximately 21% of participants were excluded, leaving 908 participants. This exclusion rate is in line with those observed in previous online studies [23]

Participants who met any one or more of the following criteria were excluded from all analyses:

- Did not provide sex assigned at birth (n = 9, <0.01%).
- Below- or near-chance task performance (overall accuracy on first decision of the information-seeking task <55%) (n = 220, 19.03%).
- Incorrect response to a ‘catch’ item employed as an attention check (n = 20, 1.73%). The ‘catch’ item was embedded within the reduced Zung Depression Scale items and read as follows: “If you are paying attention, please select ‘Good part of the time’ for this answer”.
- Used the same single confidence rating across all trials on the first decision of the task (n = 15, 1.30%).

### Statistical analyses

Paired-samples t-tests were employed to test for differences in task measures (type-1 sensitivity (***d’***), average confidence ratings, percentage of information seeking trials) between conditions. We built linear regression models to examine the relationships between task measures and psychiatric symptoms, whilst always controlling for age and sex. All regressions were conducted using the *fitlm* function in MATLAB (Mathworks, USA). Z-scores of all regressors were calculated to ensure comparability of regression coefficients. Sex was coded as female: -1, male: 1. For the regression models assessing relationships between the psychiatric symptom dimensions and the task measures, all symptom dimensions were entered in the same regression model using the following *fitlm* syntax:

$${Dependent\; Variable}\sim 1+{zscore}({AD})+{zscore}({CIT})+{zscore}({SW})+{zscore}({Age})+{Sex}$$

### Mediation analysis

This analysis was conducted using the *mediation* function (with default options) from the Mediation Toolbox (https://github.com/canlab/MediationToolbox) [40–42].

### Trial-by-trial modelling of information search

To further investigate how individuals’ confidence and the cost of information influenced seeking behaviour, we modelled information seeking on a trial-by-trial basis within participants. Taking a similar approach to Schulz et al. [8], we calculated logistic regression coefficients within each participant from a model in which a trial-wise vector of z-scored first decision-confidence judgements and the binarized cost conditions (high cost (20 points) = 1, low cost (5 points) = -1) predicted single-trial information seeking (seek = 1, not seek = 0) choices using the following model in Matlab’s *fitglm* function with the *Distribution* option set to *binomial*.

$${info\_seeking\_choice} \sim 1+{zscore}({confidence\_rating}1)+{cost\_condition}$$

From this, we were able to derive, for each participant, an intercept representing their overall willingness to seek information, a confidence coefficient representing the extent to which confidence influenced information seeking and a cost coefficient representing the influence of the cost condition on information seeking.

We then regressed these coefficients against the symptom dimension scores whilst controlling for age and sex. For this analysis, we excluded any participants who sought information on more than 95% or fewer than 5% of trials, or for whom the logistic regression model failed to converge (resulting in an N of 608).

### Multiple comparison correction

We used the Benjamini and Yekutieli (2001) procedure [43, 44] for controlling the false discovery rate (FDR) for each family of tests (specified in the results section). This provides a critical p-value according to which all uncorrected p values less than or equal to are considered significant. The desired false discovery rate was set to q = 0.05.

## Results

In an online study, participants (N = 908) from the general population completed a battery of self-report psychiatric symptom questionnaires and performed an information-seeking task with confidence ratings in which information cost (high versus low) and reward valence (appetitive versus aversive) were manipulated between blocks (see Fig. 1). Sample distributions of transdiagnostic dimension scores, tasks measures and demographics are shown in Supplementary Fig. S1.

### Adaptive use of information seeking

**Fig. 2:**
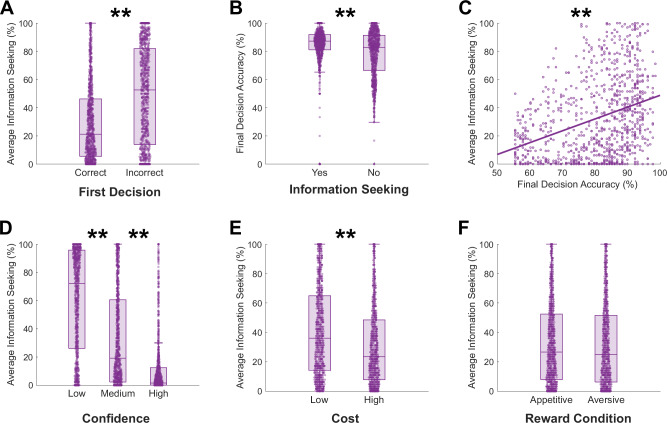
Overall use of information seeking in the perceptual task. **A** Participants were more likely to seek information after an incorrect initial decision. **B** Participants were more likely to be correct in the final decision after choosing to see the additional information. **C** Participants who sought information more often had higher overall accuracy on the final decisions. **D** Participants used the information seeking option more when their initial decision confidence was lower. **E** Participants were more likely to seek information when the cost was low. **F** There was no difference in overall information seeking between appetitive and aversive trials. ** p <.001. Together, these results show participants were using the option to seek information in a consistent and adaptive way.

First, we investigated whether participants used the opportunity to seek information in the task in an adaptive way (see Fig. 2). As expected, and in line with previous research [8], participants were more likely to seek information on trials where they initially gave an incorrect response (Fig. 2A: t(907) = –32.2, p < 0.001), and final decision accuracy was higher for trials in which participants chose to seek information than for trials in which they chose not to (Fig. 2B: t(814) = 7.70, p < 0.001).

Between-participants, those who used the opportunity to seek information more often tended to be more accurate in their final decisions (Fig. 2C: r(906) = 0.34, p < 0.001). Additionally, participants were more likely to seek information when they reported low confidence in the initial decision (Fig. 2D: low versus medium confidence (t(890) = 29.6, p < 0.001), medium versus high confidence (t(898) = 25.1, p < 0.001)) and when the cost of seeking information was low (Fig. 2E: t(819) = 15.9, p < 0.001). Finally, participants were equally likely to seek information on appetitive and aversive trials (Fig. 2F: t(907) = 0.93, p = 0.35).

### Information seeking and psychiatric symptom dimensions

**Fig. 3:**
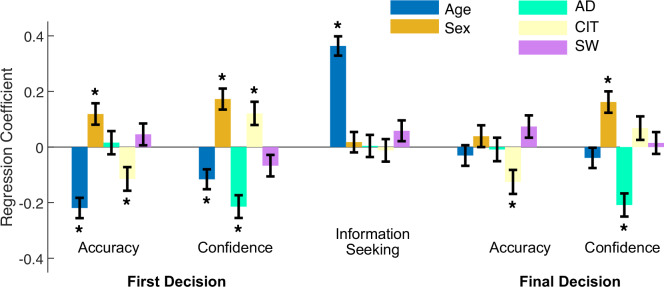
Relationships between behavioural measures and transdiagnostic symptom dimensions. Standardised regression coefficients (error bars show ± standard error) from linear models performed for each task measure (dependent variable) with age, sex (male: 1, female: -1), and symptom dimensions as independent variables. *q < 0.05 corrected for false discovery rate (FDR) over all coefficients (i.e., 5 models x 5 predictors = 25 inferences).

We next turned to our main hypotheses and investigated the relationships between the task measures, including confidence and information-seeking, and three previously identified transdiagnostic symptom dimensions of ‘anxious depression’ (AD), ‘compulsive behaviour and intrusive thought’ (CIT) and ‘social withdrawal’ (SW) [15, 17, 22, 38, 45], along with age and sex (see “Methods”). Figure 3 plots standardised regression coefficients indexing the strength and direction of the relationships between symptom dimension scores and each task measure for both the 1st and final decisions. We controlled the False Discovery Rate (FDR) at *q* = *0.05* over the 25 inferences considered (5 models x 5 predictors).

For first decisions, the accuracy and confidence results closely replicate our previous study (Benwell et al. [15]; see also Hoven et al. [16]). The CIT dimension showed a paradoxical dissociation, being significantly associated with low objective accuracy (β = –0.11, p < 0.01) but high subjective confidence (β = 0.12, p < 0.01). Conversely, the AD dimension was not associated with objective accuracy (β = 0.02, p = 0.71), but was associated with systematically low confidence (β = -0.21, p < 0.001). Interestingly, demographic variables also showed systematic relationships with first decision accuracy and confidence: older age was associated with both reduced accuracy (β = –0.22, p < 0.001) and reduced confidence (β = –0.12, p = 0.001), whereas male sex was associated with increased accuracy (β = 0.12, p = 0.002) and increased confidence (β = 0.17, p < 0.001).

Despite replicating distortions of confidence in both AD and CIT, we observed no systematic relationships between symptom dimension scores and individual differences in the total amount of information seeking (AD: β = 0.00, p = 0.92; CIT: β = -0.01, p = 0.76; SW: β = 0.06, p < 0.12). Hence, the systematic under- and over-confidence observed for first decisions in AD and CIT, respectively, did not result in systematically increased or decreased information seeking behaviour, contrary to our hypotheses. Accordingly, for the final decisions (Fig. 3B) AD remained associated with low confidence (β = –0.21, p < 0.001) and CIT was again associated with low objective accuracy (β = −0.13, p = .003), though the relationship with high confidence was no longer significant (β = 0.07, p = 0.11). Hence, individuals scoring highly for CIT did not use the information seeking option more often to improve their performance on the final decision and those scoring highly for AD did not use more information seeking to increase their confidence from the first to the final decisions.

Age was associated with reduced accuracy and lower confidence in the first decision. In contrast to AD and CIT, however, age was also associated with increased information seeking (β = 0.36, p < 0.001). Interestingly, for the final decisions (Fig. 3B) older participants no longer showed any accuracy detriment (β = –0.03, p = 0.42) or reduction in confidence (β = –0.04, p = 0.29). This indicates that, unlike for CIT and AD, older participants may have used the option to seek information to both improve performance and increase confidence in their final decisions.

One possible explanation for the positive relationship between age and information seeking may be that reduced first-decision accuracy led older participants to seek information more often than young participants. To establish if the relationship could be explained entirely by the reduced initial accuracy of older participants, we modelled information seeking with age, sex and first-decision accuracy as predictors. First-decision accuracy was related to information seeking (β = 7.46 ± 1.94 [SE], p < 0.001), but increasing age remained significantly related to increased information seeking (β = 0.71 ± 0.06 [SE], p < 8e-32) even when accounting for accuracy in the model. The fact that age predicted information seeking independently of first-decision accuracy suggests that older participants had an increased tendency to use the information seeking option in general, not just when they were initially incorrect. Indeed, age was significantly related to information-seeking both for correct (β = .36, p < 0.001) and incorrect (β = .27, p < 0.001) first-decision trials. This increased tendency to seek information regardless of initial accuracy suggests that older participants’ introspection was not necessarily optimal but rather likely reflects the fact that they had lower confidence in general about their ability to perform the task. Additionally, the increased information-seeking as a function of age was unlikely to be explained by the information being inherently more helpful for older versus younger participants (see Supplementary Fig. S2). We also investigated the relationship between age and information seeking separately for younger vs older participants (approximate median split at age=32) and found that the relationship was highly consistent in both sub-groups (Supplementary Fig. S3).

There was no relationship between sex and information seeking (β = 0.17, p = 0.63). While the association with confidence remained in the final decision (β = 0.16, p <0.001), the association with accuracy did not (β = 0.04, p = 0.32). Thus, while females did not use the information seeking option more on average, they may have used the information seeking option more efficiently to increase performance.

Note that all relationships reported above between symptom dimensions or demographic variables and task measures were replicated across all task conditions—i.e., were independent of information costs and reward valence (see Supplementary Fig. S4). We also validated that the results were not influenced by the participant exclusion criteria, by repeating the analysis shown in Fig. 3 on the full dataset with no participants excluded (Supplementary Fig. S5). In line with previous studies [12, 15, 16, 46], no relationships surviving multiple comparison correction were found between metacognitive efficiency (meta-d’/d’) and any of the symptom dimensions, age, or sex for either the first or final decisions (see Supplementary Fig. S6).

### Information-seeking mediates age-related increases in accuracy and confidence from first to final decisions

**Fig. 4:**
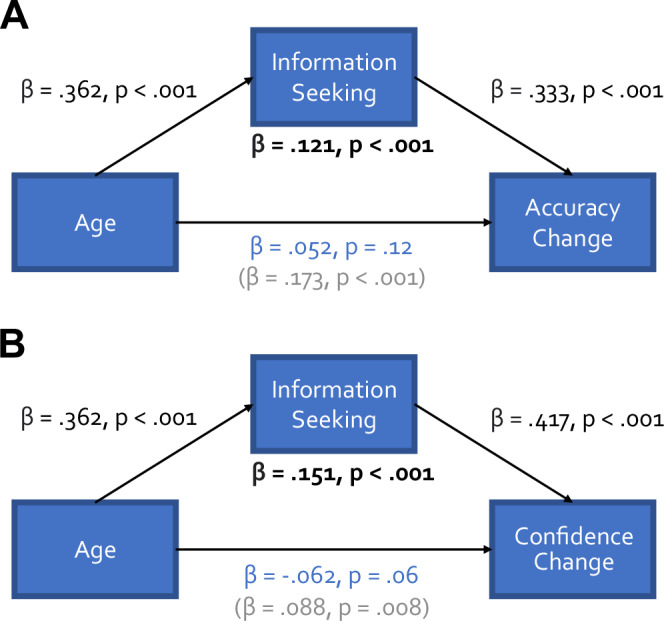
Mediation analysis of age-related information seeking effects. Increased information seeking mediated a relationship between age and changes in accuracy between first and final decisions (**A**), and between age and changes in confidence (**B**). The mediated relationships are shown in bold; the unmediated relationships are shown in blue, and the total relationships are shown in light grey.

Having established older adults sought more information overall, we next tested whether this had resulted in increases in either accuracy and/or confidence in their final decisions using mediation analyses (Fig. 4). Older participants more often changed from inaccurate first responses to accurate final responses (total effect: β = 0.173, p < 0.001) and this effect was fully mediated by their increased willingness to seek information (mediation effect: β = 0.121, p < 0.001, corrected direct effect: β = 0.052, p = 0.12) (Fig. 4A). Additionally, older participants increased in confidence more from first to final decisions (total effect: β = 0.088, p = 0.008) and this effect was also fully mediated by their increased willingness to seek information (mediation effect: β = 0.151, p < 0.001, corrected direct effect: β = –0.062, p = 0.06) (Fig. 4B).

Together, these findings show that increased information seeking in older participants improved their objective perceptual performance and increased their confidence accordingly.

### Trial-by-trial modelling of information search

**Fig. 5:**
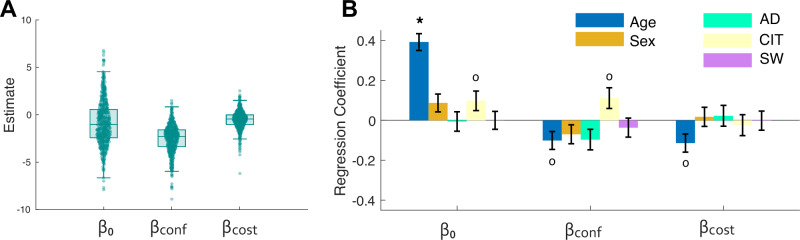
Trial-by-trial logistic regression model of information seeking behaviour. **A** Boxplots showing the distributions of within-participant logistic model coefficients. The decision to seek additional information was captured using a model with three parameters: an intercept β_0_, a confidence parameter β_conf_, and a cost parameter β_cost_. **B** Standardised regression coefficients (error bars show ± standard error) from linear models applied to each coefficient (dependent variable) with age, sex, and symptom dimensions as independent variables. °p < 0.05 uncorrected; *q < 0.05 corrected for false discovery rate (FDR) over all coefficients shown (i.e. 3 models x 5 predictors = 15 inferences).

Finally, we investigated how trial-by-trial decisions to seek information were informed by initial confidence judgements and the cost of information within participants [8]. Using a logistic regression model predicting single-trial choices to seek information from initial confidence ratings and information cost (–5 or –20 points) (see “Methods”), we derived measures for each participant indexing three independent behavioural phenomena: an intercept (β_0_) representing a general shift in willingness to seek information, and coefficients representing how strongly initial confidence (β_conf_) and information cost (β_cost_) influenced choices to seek information. The distributions of the coefficients are shown in Fig. 5A. We then regressed these measures against the demographics and symptom dimensions (see Fig. 5B).

As expected, based on the model-free results reported in Fig. 3 and given that β_0_ captures the overall average information seeking, we found no significant relationships surviving multiple comparison correction between the intercept (β_0_: indexing the overall tendency to seek information) and any of the symptom dimensions (Fig. 5A) (AD: β = –0.0006, p = 0.90; CIT: β = 0.098, p = 0.046; SW: β = 0.00, p = 0.99). Additionally, the single-trial modelling revealed no differential influence of confidence (β_conf_) (AD: β = –0.09, p = 0.06; CIT: β = 0.11, p = 0.03; SW: β = –0.03, p = 0.44) or cost (β_cost_) (AD: β = 0.02, p = 0.66; CIT: β = –0.02, p = 0.65; SW: β = –0.00, p = 0.97) on information seeking for any of the symptom dimensions.

In line with the model-free results, age was associated with an increased overall tendency to seek information (intercept: β = 0.39, p < 0.001). However, relationships between age and the influence of initial confidence, β_conf_ (β = –0.10, p = 0.02) and cost β_cost_, (β = –0.11, p = 0.01) did not survive control for False Discovery Rate. Together, this indicates that older participants were not more influenced by their initial confidence or the cost of seeking when deciding whether to seek information compared with younger participants.

## Discussion

Distortions of confidence have been linked to both aging [19–21] and various forms of psychopathology [13, 14] but it remains unknown whether confidence-related behaviours such as information seeking are affected. Here, confidence abnormalities associated with distinct transdiagnostic symptom dimensions (low confidence in anxious-depression and impaired accuracy/overconfidence in compulsivity) did not result in altered information-seeking prior to final perceptual decisions. In contrast, older participants displayed an adaptive use of information-seeking, whereby they gathered additional perceptual information to overcome initial objective accuracy and confidence deficits.

In line with previous research, an ‘anxious-depression’ (AD) dimension was associated with low confidence in task performance, even in the absence of objective deficits [12, 15–17, 47]. However, when high AD participants had the chance to increase confidence through seeking additional information, they did not take it more often than those scoring low for AD. Hence, high AD participants remained low in confidence in their final decisions. These results are in line with previous studies which have not found an overall increased tendency to seek information in depression [48] or anxiety [49] and suggest that the low confidence experienced by AD individuals does not drive increased information seeking. This apparent dissociation between confidence and information-seeking may be explained by high AD participants anticipating that information seeking would not effectively reduce their uncertainty and may even add to it [48, 50, 51]. Alternatively, anhedonia and apathy represent cardinal symptoms of depression [52, 53] which may reduce the motivation of high AD participants to increase their confidence through actively seeking information.

The ‘compulsive behaviour and intrusive thought’ (CIT) dimension was associated with reduced 1^st^-order accuracy but a paradoxical positive confidence bias, in line with previous research [15–17, 54, 55]. However, CIT was not associated with altered information-seeking, and high CIT participants remained impaired in their final decisions. Hence, high CIT participants did not use the opportunity to seek information enough to overcome their initial accuracy detriment, potentially due to inappropriately high confidence. Interestingly, Hauser et al., [56] found that when there was no cost to information-seeking, highly compulsive individuals sought more information than low compulsive individuals prior to committing to a final decision. However, when there was a cost to information-seeking, no difference was observed between high and low compulsive groups, in line with the current study. Future studies could investigate further how cost-benefit trade-offs influence information-seeking in compulsive individuals [57].

Our results provide further evidence for an altered mapping between confidence and behaviour in compulsive individuals [55], which may impair decision-making and contribute to cognitive inflexibility [58, 59]. It remains unclear exactly what causes the 1st-order decision deficits associated with CIT. In our previous study [15], we observed CIT-related decision impairments across both perceptual and semantic knowledge tasks, thereby ruling out an explanation in terms of low-level sensory dysfunction. Alternatively, the decision impairments may arise due to some combination of increased choice variability/choice history bias [59–61], inappropriate speed-accuracy trade-off [62], and/or a recently proposed ‘decision acuity’ trait found to underlie performance across a range of decision tasks [63].

By employing a low-level perceptual decision-making task, we were able to rule out any potential influence of contextual factors, motivated reasoning and/or prior knowledge on task performance [8, 18]. This allowed us to test whether symptom-related signatures of confidence and/or information-seeking exist as core cognitive traits, potentially influencing decisions related to all aspects of a person’s life, not just those directly related to their symptoms. Based on our results, we propose that impaired decision-making (in CIT) and confidence alterations (low confidence in AD and high confidence in CIT) appear to represent core cognitive traits associated with symptomology, whereas systematically altered information-seeking does not. However, this does not rule out the possibility that symptom-related information-seeking alterations do exist in specific contexts and domains. For instance, anxious individuals may selectively increase information-seeking when faced with real-world threats and/or when they experience large changes in their external environment [49]. Additionally, health anxiety is known to be associated with excessive searching for health-related information [64, 65]. Hence, psychiatrically relevant information-seeking behaviours are unlikely to represent a core computational deficit but rather selective alterations for stimuli related to symptom-relevant themes.

In contrast to the symptom dimensions, we observed increased use of information-seeking related to aging. Older adults performed objectively worse in their initial perceptual decisions [66–69] and reported lower confidence. However, they improved their accuracy and confidence through increased information-seeking and performed just as well as younger adults for final decisions. These results are in line with a well-preserved metacognitive capacity in older age [20, 70, 71] as older individuals appropriately operationalised their uncertainty in the form of information seeking [1], in contrast to individuals reporting high levels of AD and CIT. An important step in future studies will be to ascertain whether the age-related increase in information-seeking observed here generalises to different tasks and to alternative forms of information manipulations. For instance, the manipulation of evidence strength when seeking extra information used in the current study was an increased duration of stimulus presentation time, which may have been particularly favourable for older participants. It remains to be seen if similar results would be seen for alternative manipulations such as an easier version of the stimulus being presented for the same fixed presentation time.

Information-seeking represents one of many behaviours strongly influenced by subjective confidence. Future studies can investigate whether age and psychiatrically relevant confidence changes influence other confidence-related behaviours such as deliberation [72], cognitive offloading [73–75], learning [5] and/or the engagement of cognitive control [76, 77]. Here, we adopted a transdiagnostic, dimensional approach to symptoms and assessed variation in the general population [38, 78]. It would also be of interest to test confidence-behaviour relationships in clinical samples with high levels of symptom severity. Understanding the behavioural signatures of psychiatric symptoms can help to identify factors which perpetuate poor mental health and potentially inform intervention and prevention.

## Supplementary information

Supplementary Information

## Data Availability

All data are openly available on the Open Science Framework (OSF) under the URL: https://osf.io/wamgt/.
