# Supplementary material for: Information search under uncertainty across transdiagnostic psychopathology and healthy ageing
Source: Transl Psychiatry. 2024 Sep 3;14:353. doi: 10.1038/s41398-024-03065-w (PMC11372192; doi:10.1038/s41398-024-03065-w)
Supplement: Supplementary file 1 — Supplementary Information [file 41398_2024_3065_MOESM1_ESM.docx]

# **Supplementary Information**

# **Information search under uncertainty across transdiagnostic psychopathology and healthy ageing**

Greta Mohr^1^, Robin A. A. Ince^1,*^ , Christopher S.Y. Benwell^2,*^

^1^ School of Psychology and Neuroscience, University of Glasgow; Glasgow, UK.

^2^ Division of Psychology, School of Humanities, Social Sciences and Law, University of Dundee; Dundee, UK.

^*^ - equal contribution

Corresponding author: c.benwell@dundee.ac.uk

# **Supplementary Figures**


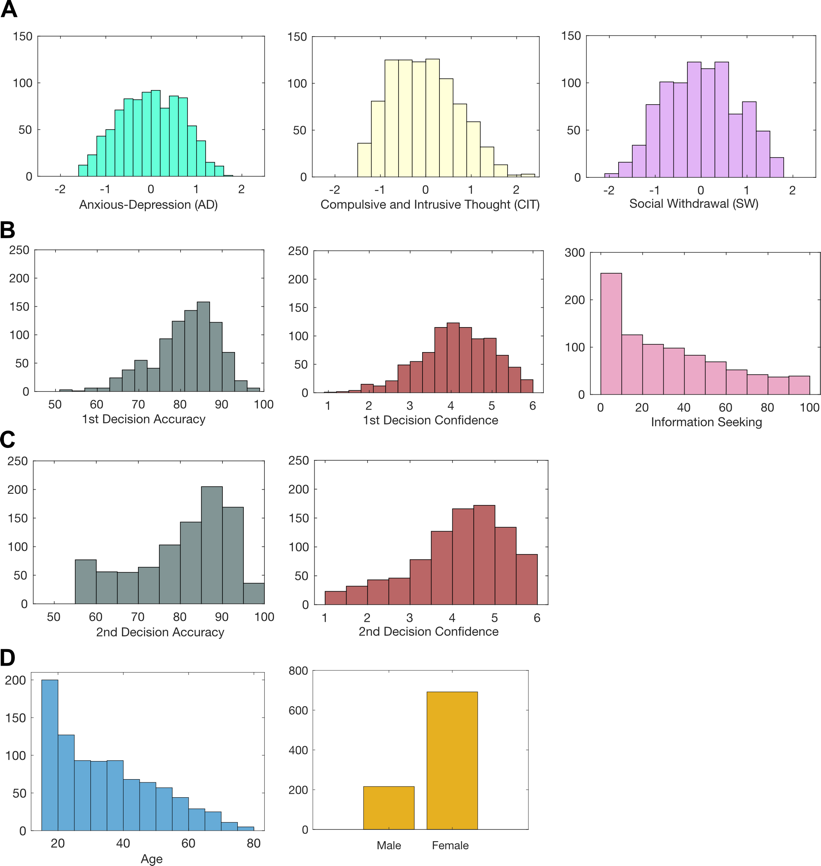


**Figure S1.** *Experiment data and demographics.* **A**: Distributions of transdiagnostic symptom dimension scores. **B**: Distributions of first decision task measures (collapsed across all levels of perceptual evidence). **C**: Distributions of final decision task measures (collapsed across all levels of perceptual evidence). **D**: Distributions of demographics.


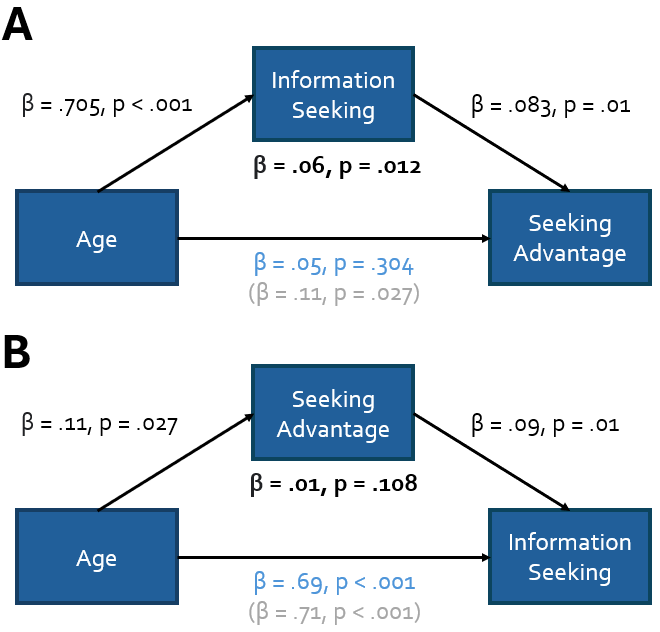


**Figure S2.**  *Mediation analyses of age-related information seeking advantage*. A potential explanation for the age-related increase in information seeking may have been that the experimental manipulation was inherently more helpful for older participants. This interpretation is in line with the fact that the advantage gained from seeking information (calculated as ‘Final decision accuracy on seeking trials’ - ‘Final decision accuracy on non-seeking trials’) increased as a function of age (β = 0.05, p = .027). At face value, this result is difficult to interpret. We know that older participants used the information seeking more often (as shown in our main analyses) and so this may explain the age-related increasing advantage effect (i.e., using the information seeking option more often may have led to a bigger difference in final decision accuracy between seeking and non-seeking trials). However, an alternative possibility is that the extra information presented in this paradigm was inherently more informative for older participants. It could also be the case that both factors contribute. Hence, these hypotheses can be summarised as follows:

**A)** Older participants sought information more often and accordingly benefitted more.

**B)** Information seeking in the task was inherently more informative for older participants.

**C)** Both factors contributed to the effect

To test these hypotheses, we performed two separate mediation analyses. In the first analysis (Figure S2A), we reasoned that if the information seeking manipulation was inherently more informative for older participants than young (Hypothesis **B**), then age should be significantly related to information seeking advantage even when the amount of information seeking is controlled for. In other words, when the amount of information seeking is controlled for, older participants should still show an increased information seeking advantage. Alternatively, if the relationship between age and information seeking advantage is explained by older participants using the information seeking option more often (Hypothesis **A**), then the relationship between age and information seeking advantage should be weakened (Hypothesis **C**) or eliminated (Hypothesis **A**) when the amount of information seeking is controlled for. We tested the hypotheses with a mediation model in which the ‘% of information seeking trials’ was proposed as a mediator of the ‘age’ – ‘information seeking advantage’ relationship (see Figure below). The results supported Hypothesis **A:** The increased information seeking advantage as a function of age (total effect: β = .11, p = .027) was fully mediated by the increased % of information seeking trials as a function of age (mediation effect: β = .06, p = 0.012, corrected direct effect: β = .05, p = 0.3).

In a second analysis (Figure S2B), we further tested the concern that information seeking may have increased with age only because the information was inherently more useful for older participants with an alternative mediation model. Here, we reasoned that if the age-related increase in the amount of information seeking could be explained away by increased usefulness of information seeking with age, then the relationship between age and the amount of information seeking would be weakened or eliminated when the information seeking advantage was controlled for. We tested this hypothesis with a mediation model in which the ‘information seeking advantage’ was proposed as a mediator of the ‘age’ – ‘% of information seeking trials’ relationship (see Figure below). The results showed that information seeking advantage did not mediate the relationship between age and the amount of information seeking (mediation effect: β = .012, p = 0.108, total effect: β = .71, p < .001, corrected direct effect: β = .694, p < .001).

Taken together, the evidence from the two mediation models supports an interpretation that the age-related increase in information seeking cannot be explained away by the experimental manipulation only being helpful in older participants. We note that in the youngest participants (aged 17-22) there was already a clear advantage in final decision accuracy for seeking versus non-seeking trials (t(261) = 2.83, p=0.005), thereby showing that the manipulation was helpful even in the youngest participants in the sample.


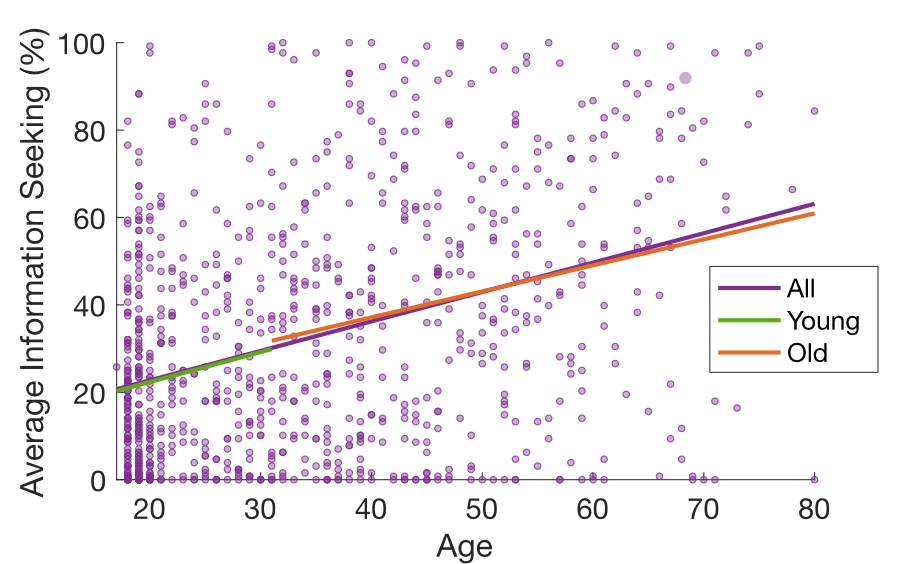


**Figure S3.** *The relationship between age and information seeking is consistent across the sampled range of ages.* Scattered points show the relationship between age (x-axis) and average information seeking (y-axis) across participants (points). We fit three linear regression models to this relationship. The first model uses all participants (purple line; β = 0.67 ± 0.06 [SE], p < 2e^-29^). We then median split the data and fit the same linear model to younger (age <= 31, N = 459, β = 0.68 ± 0.25 [SE], p < .008) and older (age > 32, N = 449, β = 0.59 ± 0.12 [SE], p < 2e^-6^) participants separately. The relationship between age and information seeking was consistent between these models.

**Figure S4.** *Replication of overall findings in each task condition*. Bar charts show the standardised regression coefficients (error bars show +/- standard error) from a linear model for each task measure (dependent variable) with age, sex, and symptom dimensions as independent variables. **A**: First decision accuracy. **B**: First decision confidence. **C**: Information seeking. **D**: Final decision accuracy. **E**: Final decision confidence. *p < 0.05. We found that all relationships were consistent with the main findings across all conditions in terms of significance and direction of effect.

**Figure S5**. *Results are not sensitive to participant exclusion criteria*. To validate the robustness of our key results (Figure 3), we repeated the analysis without any participant exclusions. The pattern of the key results is highly consistent (all significant results in Figure 3 remain significant).

**Figure S6.** *No significant relationships with metacognitive efficiency (M-ratio)*. Standardised regression coefficients (error bars show +/- standard error) from a linear model for first and final decision metacognitive efficiency (dependent variable) with age, sex, and symptom dimensions as independent variables. °P < 0.05 uncorrected. No coefficients survive correction for false discovery rate (FDR) over all coefficients shown (i.e., 2 models x 5 predictors = 10 inferences).
